# Network reconfiguration and DG based compensation of Wolaita Sodo distribution system by using particle swarm optimisation

**DOI:** 10.1371/journal.pone.0335512

**Published:** 2025-10-30

**Authors:** Biniam Alemayehu, Satyasis Mishra, Ghanshyam G. Tejani, Sujan Tripathi

**Affiliations:** 1 Department of Electrical and computer engineering, University of Wolaita Sodo, Sodo, Ethiopia; 2 Department of ECE, Centurion University of Technology & Management, Bhubaneswar, Odisha, India; 3 Department of Research Analytics, Saveetha Dental College and Hospitals, Saveetha Institute of Medical and Technical Sciences, Saveetha University, Chennai, India; 4 Department of Industrial Engineering and Management, Yuan Ze University, Taoyuan, Taiwan; 5 Institute of Engineering (IoE), Thapathali Campus, Tribhuvan University, Kathmandu, Nepal; Southwest University of Science and Technology, CHINA

## Abstract

Improving voltage profiles and reducing power losses are critical challenges in modern distribution systems, especially in real, unbalanced and weakly interconnected grids. This study presents a novel application of simultaneous network reconfiguration and distributed generation (DG) placement using Particle Swarm Optimization (PSO) to the Wolaita Sodo distribution network in Ethiopia. Unlike most prior works conducted on IEEE standard feeders, this research uses actual network topology, load data, conductor parameters and operational constraints from a real Ethiopian distribution system. The optimization problem considers radiality constraints, DG size limits and voltage and current limits, with objectives of minimizing active/reactive power losses, improving voltage profiles and maximizing economic benefits. A backward-forward sweep load flow method evaluates network performance under four scenarios: base case, reconfiguration only, DG placement only and simultaneous implementation. Results show that the proposed method reduces active power losses by 72.064%, decreases voltage deviation from 24.63% to 4.5% and improves the minimum bus voltage from 0.7537 pu to 0.9550 pu. The annual financial savings of 16.3956 million ETB yield a payback period of six years. This grouping of real-network data, simultaneous optimization, and integrated techno-economic analysis offers a practical and replicable decision-support tool for utilities in emerging power systems.

## 1. Introduction

The continuous improvement of living conditions around the world is greatly impacted by electricity, which has become a necessary component of our daily lives and is vital for industrial development. Electricity demand continues to rise as it powers all electrical equipment. In a power system, distribution, comprising of low-voltage cables and transformers is the largest and final stage of supply. Feeders are equipped with sectionalising switches (normally closed) and tie switches (normally open) to enhance reliability and maintain loads during failures. Network reconfiguration, first introduced by Merlin and Back in 1975, refers to adjusting these switches to reduce line losses under specific load conditions [1]. By modifying the open/close status of switches, Distribution Feeder Reconfiguration (DFR) modifies the feeder circuit topology in electrical distribution networks. DFR helps to improve overall system performance, lower energy losses, and increase distribution system efficiency by dynamically optimising feeder circuits, particularly when it comes to load management and the integration of renewable energy [2]. Several researchers have studied DG placement problem using a different techniques, and one of such as Particle Swarm Optimization. The stochastic optimisation technique known as Particle Swarm Optimisation (PSO) was first presented by Drs. Eberhart and Kennedy in 1995. It was inspired by the collective behaviour seen in fish schools and flocks of birds [3,4].

The main objectives of distribution system reconfiguration are to improve voltage profiles and reduce power losses. Power flow analysis is therefore essential for evaluating the steady-state electrical characteristics brought forth by reconfiguration. Although Gauss-Seidel, Newton-Raphson (NR), and their decoupled counterparts are frequently used in power flow analysis procedures for gearbox systems, these methods presume a balanced system by representing three-phase systems in a single phase. However, a different strategy is required for distribution systems, which are distinguished by scattered generation, radial topology, unbalanced loads, and frequently un-transposed lines.

A Case Study

**Fig 1:**
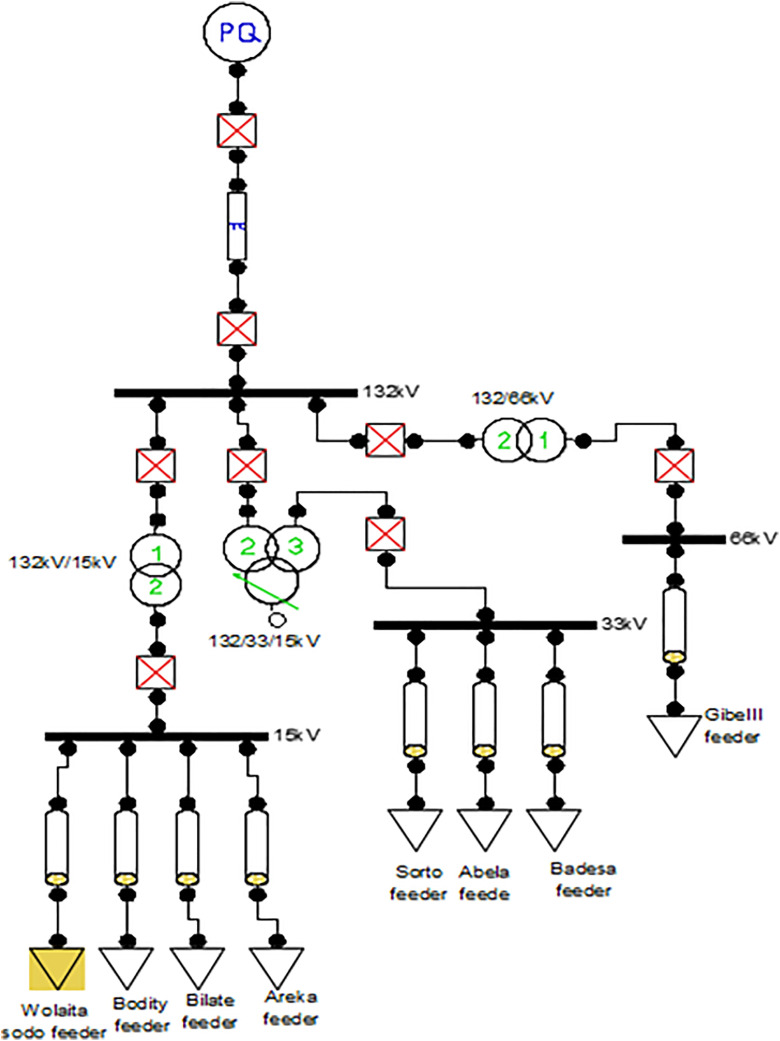
Wolaita Sodo Distribution Substation.

The Wolaita Sodo Distribution Substation is configured with one incoming feeder and eight outgoing feeders shown in Fig 1.

- The incoming feeder operates at 132 kV, receiving its supply from the main interconnected system (ICS) through the Lasho Transmission Grid, which operates at 400 kV.
- The eight outgoing feeders are categorized based on their voltage levels:
  - One 66 kV feeder
  - Three 33 kV feeders
  - Four 15 kV feeders

The outgoing feeders from the Wolaita Sodo Substation are designated as follows:

- 66 kV Gibe III Feeder
- 15 kV Wolaita Sodo Feeder
- 15 kV Bodity Feeder
- 15 kV Areka Feeder
- 15 kV Bilate Feeder
- 33 kV Abala Feeder
- 33 kV Bedessa Feeder
- 33 kV Bale Sorto Feeder

This configuration ensures efficient distribution of power across various regions, supporting both high-voltage and medium-voltage networks in the service area. The feeder highlighted in yellow in the figure below represents the specific area where this study or project is being implemented. This feeder, located within Wolaita Sodo town, is connected to a network of 114 distribution transformers, collectively providing an installed capacity of approximately 27 MVA. The peak power demand recorded for the town ranges between 10.98 MW and 12 MW, reflecting the growing energy requirements of the region. The town’s distribution network is constructed using bare conductors of type AAC95 and AAC50, which are widely used for medium- and low-voltage power distribution.

The overhead distribution lines extend across a total length of approximately 56 kilometers, ensuring the delivery of electrical energy throughout the urban area.

The suggested work is divided into the following sections: The introduction is in Section I, followed by the Literature survey in Section 2, methodology and formulation of the problem in Section 3, the results and discussion in Section 4, and the conclusion in Section 5.

This paper proposes a different case.

Case 1: Base Case

Case 2: System with reconfiguration only

Case 3: System with DG placement only

Case 4: System with Simultaneous Network Reconfiguration and DG Allocation

B Power Flow Analysis

In electrical engineering, power flow analysis, also known as load flow analysis, is a crucial study that establishes how electric power is distributed in a power system under steady-state circumstances. It seeks to determine power losses in the system, evaluate the flow of active and reactive power from generators to loads, and compute the voltage levels at different locations throughout the network. Understanding voltage profiles, organising system expansions, guaranteeing operational dependability, and improving power flow control techniques all depend on this analysis. The Newton-Raphson, Gauss-Seidel, and Fast Decoupled methods are frequently used techniques for power flow analysis; their efficiency and complexity vary according to the features of the power system under study. Distribution systems, characterized by unbalanced loads, radial topology, often un-transposed lines, and distributed generation, necessitate a different approach. Therefore, a power flow method accommodating three-phase unbalanced networks is imperative for distribution systems. Backward-Forward methods, a popular choice for power flow analysis in distribution systems.

C **Network Reconfiguration**

Network reconfiguration in power systems involves adjusting the configuration of the electrical distribution network by changing the status of switches, breakers, and control devices with the aim of optimizing system operation. The primary objectives include minimizing power losses, improving voltage profiles, enhancing reliability, balancing loads, and integrating distributed energy resources

D **Distribution Generation**

Distributed Generation (DG) refers to the generation of electricity from multiple small-scale energy sources that are dispersed throughout an electricity grid. Unlike traditional centralized power plants, which are large-scale facilities located in remote areas, DG systems are typically installed close to the point of electricity consumption. Distributed generation technologies include solar photovoltaic panels, wind turbines, micro-turbines, fuel cells, and small-scale hydroelectric generators, among others. implemented distributed resources to minimize power loss in a radial distribution feeder.

E Novelty of the research

The paper introduces a combined approach of network reconfiguration and DG allocation using Particle Swarm Optimization (PSO), instead of treating them separately. A Backward-Forward Sweep (BFS) load flow method is employed to handle unbalanced, radial distribution networks, which is more suitable for real distribution systems compared to conventional load flow methods. The study’s main novelty lies in its practical case-based validation (using a real Ethiopian distribution feeder), integration of reconfiguration and DG placement, and demonstrated cost-effectiveness. Unlike most prior works that use IEEE standard test feeders, this study applies network reconfiguration and distributed generation (DG) placement to the actual Wolaita Sodo distribution system in Ethiopia, using real load data, network topology, and conductor parameters. Improving voltage profiles and reducing power losses are critical challenges in modern distribution systems, especially in real, unbalanced and weakly interconnected grids. This study presents a novel application of simultaneous network reconfiguration and distributed generation (DG) placement using Particle Swarm Optimization (PSO) to the Wolaita Sodo distribution network in Ethiopia. Unlike most prior works conducted on IEEE standard feeders, this research uses actual network topology, load data, conductor parameters and operational constraints from a real Ethiopian distribution system. The optimization problem considers radiality constraints, DG size limits and voltage and current limits with objectives of minimizing active/reactive power losses, improving voltage profiles and maximizing economic benefits. A backward-forward sweep load flow method evaluates network performance under four scenarios: base case, reconfiguration only, DG placement only and simultaneous implementation. Results show that the proposed method reduces active power losses by 72.064%, decreases voltage deviation from 24.63% to 4.5% and improves the minimum bus voltage from 0.7537 pu to 0.9550 pu. The annual financial savings of 16.3956 million ETB yield a payback period of six years. The proposed approach reduces annual energy losses from 16.669 GWh to 4.1643 GWh, leading to savings of 16.3956 million ETB/year with a payback period of 6 years.This combination of real-network data, simultaneous optimization and integrated techno-economic analysis offers a practical and replicable decision-support tool for utilities in emerging power systems.

The contributions are mentioned as follows

This work differs from prior PSO-based reconfiguration and DG studies in three ways:

i It targets a real utility-scale ethiopian feeder (wolaita sodo) with detailed unbalanced three-phase modelling and bfs load flow rather than benchmark ieee test feeders ensuring practical fidelity for aac-95/aac-50 conductors and field transformer loading.
ii It solves switch status and multi-dg sizing/placement simultaneously under a single objective with explicit radiality, current, voltage and power-balance constraints implemented with a PSO search tailored to the feeder’s minimal-cutset space.
iii It provides a cost/benefit analysis with payback estimation to support investment decisions. on the wolaita sodo feeder the simultaneous scheme achieves 72.064% active-loss reduction, improves the minimum voltage from 0.7537 pu to 0.9550 pu and lowers voltage deviation from 24.63% to 4.5% outperforming either reconfiguration-only or DG-only strategies, and yielding ETB (Ethiopian Birr) 16.3956 million/year savings with a ~ 6-year payback.

## 2. Literature survey

Lottif et al. in 2024: In order to lower power losses, Merlin first suggested distribution network reconfiguration (DNR) in 1975, using the brunch and bind algorithm to solve the dc load flow reconfigrution problem [5]. Fakir et al in 2024: presents an enhanced Backward/Forward Sweep (BFS) algorithm for load flow calculations in radial distribution systems, addressing convergence issues in traditional methods like Newton-Raphson and Gauss-Seidel. It improves accuracy by using precise voltage drop formulas in line with IEEE standards and considers various load types [6]. Murari et al. [7] in 2023proposed a Backward-Forward Sweep (BFS) algorithm for power flow analysis in AC-DC distribution networks, by accommodating various distributed generation models and converter operation modes [7]. T.Deosaria et al. [8] introduced the backward-forward sweep method for load flow analysis in radial distribution systems. Choobdari et al. [9] proposed a two-level optimization model aimed at enhancing the resilience and efficiency of smart distribution networks through dynamic reconfiguration. Balmukund kumar et al. [10], 2020, conducted optimal reconfiguration of the distribution network to improve network efficiency using a multi-objective Particle Swarm Optimization (PSO) approach.. Yhiaoui Merzoug et al. [11] proposed optimizing the reconfiguration of distribution feeders to minimize losses using the Particle Swarm Optimization (PSO) method. A.S.Abubakar et al. [12] in 2019: presented a method employing the firefly algorithm (FA) to identify the best sequence of switching operations for minimizing daily power loss and improving voltage profiles. A.Ebrahimi et al. [13] in 2024, focussed study on the optimal allocation and sizing of Distributed Static Synchronous Compensators (DSTATCOM) and photovoltaic Distributed Generation (PV-DG) units in distribution networks, addressing uncertainties in generation and consumption. An improved Teaching-Learning-Based Optimization (TLBO) algorithm to enhance voltage profiles and minimize power losses [13]. Yuvaraj T et al in 2023: The study investigates the optimal allocation and sizing of Distributed Static Compensators (DSTATCOM) [14], photovoltaic Distributed Generation (PV-DG), and Battery Energy Storage Systems (BESS) in radial distribution systems, particularly in the context of integrating Electric Vehicle Charging Stations (EVCS). Y.Werkie and Yalew Gebru in 2022, investigated optimal allocation of multiple distributed generation (DG) units in power distribution networks to improve voltage profiles and minimize power losses. Utilizing an improved particle swarm optimization (IPSO) methodology, the research demonstrates significant reductions in power losses [15]. Mandefro Elias in 2020: study focused on enhancing the performance of a radial distribution network through the optimal placement of distributed generation (DG). The author aimed to minimize power loss, reduce interruptions, and lower costs [16]. S.Kamel et al. [17] in 2023: presented a novel approach for simultaneously optimizing the allocation of Distributed Generation (DG) units and network reconfiguration (NR) in distribution networks using the Geometric Mean Optimization (GMO) algorithm combined with a Power Loss Sensitivity Index (PLSI). Muqthiar Ali Shaik et al in 2022: Discusses the use of Equilibrium Optimizer (EO) for reconfiguring distribution networks and optimal placement of Distributed Generation (DG) units to improve voltage profiles and reduce power losses [18]. Manikanta et al. (2025), investigated the combined implementation of distributed energy resources (DERs) and network reconfiguration with a focus on cost–benefit analysis in distribution systems [19].Waseem Haider et al. [20], in 2024 discussed the Voltage Profile Enhancement and Loss Minimization in a reconfigured network using Distributed Generation. Manikanta et al. [21], examined the impact of integrating distributed generators (DGs) into radial distribution networks under realistic load models, with a focus on improving system performance indices. The study emphasized on considering realistic, mixed load characteristics rather than idealized assumptions. Consequently, the proposed approach offers a robust framework for enhancing reliability, efficiency, and sustainability in modern power distribution networks [21].

Tekele et al. [22], addresses a the enhancement of reliability and voltage profiles in radial distribution systems through the optimal placement of Distributed Energy Resources (DERs). The authors’ focussed on two practical feeders from the Debre Markos (D/M) city distribution network in Ethiopia which provided a valuable, real-world case study. Tekele et al. [23], offers a comprehensive review of hybrid renewable energy power production systems for both on-grid and off-grid applications. The review meticulously examines the key challenges in optimally sizing hybrid renewable energy sources (HRES), detailing essential components, parameters, and methodologies. It also outlines the goal functions, design constraints, and various optimization tools and meta-heuristic algorithms used in previous studies. Isaac Amoussou et al. [24], proposed a valuable study on the replacement of light fuel oil (LFO) thermal power plants in northern Cameroon with renewable energy systems. The work proposes and evaluates several scenarios, including solar photovoltaic (PV) with pumped hydro storage (PHSS), wind with PHSS, and a hybrid PV-Wind-PHSS system. Notably, the PV-Wind-PHSS hybrid system proves to be the most economically viable option, with a total cost 4.6% and 17% less than the Wind-PHSS and PV-PHSS scenarios, respectively, at 0% LOLP, confirming the project’s profitability regardless of the chosen. Tekele et al. [25], presents a hybrid renewable energy system (HRES) comprising photovoltaic units, a biogas generator, and multiple energy storage technologies such as superconducting magnetic energy storage (SMES) and pumped hydro energy storage (PHES). The study highlights the challenges posed by fluctuating load demands,. To address this, a fractional order fuzzy-PID (FO-Fuzzy-PID) controller, optimized using the opposition-based whale optimization algorithm (OWOA), was proposed. The results of the frequency deviation obtained by using FO-fuzzy-PID controllers with OWOA tuned are 1.05%, 2.01%, and 2.73% lower than when QOHSA, TBLOA, and PSO have been used to tune, respectively.

A techno-economic feasibility study in Ethiopia compared a 500 kW diesel generator with a PV–battery priority grid-tied system for Debre Markos University, presented by Aeggegn et al. [26], where annual grid interruptions exceeded 800 hours. The analysis revealed that diesel-based generation, though widely used across universities and government agencies, is economically unsustainable beyond five years due to high fuel and maintenance costs. In contrast, the PV–battery system not only proved technically feasible but also reduced carbon emissions by 94%, underscoring its superior economic and environmental performance in a country heavily reliant on oil imports and facing limited rural electrification. A recent study by Agajie et al. [27], explored a grid-connected hybrid PV–biogas system with SMES–PHES storage to address frequent grid outages, evaluating performance in terms of economic viability, reliability, and environmental impact. Using MATLAB-based simulations, metaheuristic techniques including NSWOA, MOGWO, and MOPSO were compared, with NSWOA achieving superior results across net present cost (€6.997 × 10⁶), loss of power supply probability (0.0085), and greenhouse gas reduction (7.37 × 10⁶ kg). Amoussou et al. [28], investigated the technical and economic feasibility of replacing all heavy fuel oil (HFO) and light fuel oil (LFO) thermal power plants in southern Cameroon with solar photovoltaic (PV). Using the Multi-Objective Bonobo Optimizer (MOBO), the authors optimized both system sizing and PV site selection, The findings confirmed that the proposed hybrid system is both the most economical and environmentally sustainable compared to grid-plus-DG and DG-only scenarios [29]. An Efficient Metaheuristic BitTorrent (EM-BT) algorithm has been proposed by Fettah et al. [30], to optimize the placement and sizing of photovoltaic renewable energy sources (PVRES) and capacitor banks in distribution networks, with the objective of minimizing energy losses and improving voltage stability under varying load, irradiance, and temperature conditions. A hybrid renewable system integrating PV, wind, and battery storage was recently optimized using an ANFIS-based MPPT and ANFIS-PI controller results confirmed the approach’s effectiveness, highlighting its potential for powering remote regions [31]. An enhanced whale optimization algorithm (EWOA) was used to optimize a hybrid solar PV–biogas system integrated with SMES and PHES, aiming to improve distribution system performance by reducing outages, losses, and voltage instability. Compared with AVOA, GWO, and WCA, the EWOA achieved superior results in minimizing life cycle cost and enhancing reliability, demonstrating the effectiveness of advanced metaheuristics in sizing hybrid renewable–storage systems [32]. Badraddine et al. [33], introduced a real-time implementation of Model Predictive Control (MPC) for DC–DC boost converters under variable loading, demonstrating superior performance over traditional PI controllers by minimizing oscillations in both transient and steady-state current responses. The approach, validated experimentally on inductive and resistive loads, highlights MPC’s effectiveness in precise current regulation and duty cycle control, marking a novel contribution to converter applications. A study on PV power forecasting combined feature selection methods such as ReliefF and Chi-square with neural networks, demonstrating that careful predictor selection significantly improves accuracy and model efficiency. Using real-world data from southern Algeria, the best results were achieved with ReliefF–MLP (nMAE = 9.21%, R² = 0.9608) and Chi-square–LSTM (nMAE = 9.29%, R² = 0.946), highlighting the value of feature selection for reliable solar energy forecasting and grid stability [34]. Table 1shows the summary of some of the literature reviews.

**Table 1:** Summary of different review.

Author and year	Algorithm used	Network Type	Optimization Objective	Results	Limitations
Kumar et al. (2020)	Multi-Objective PSO	IEEE 33-bus	Loss minimization and Voltage Profile	Reduce Loss by 60%	Standard Feeder only
Kamel et al. (2023)	GMO+PLSI	IEEE 69-bus	Loss minimization and Voltage Profile	Reduce loss by 68%	
Shaik et al. (2022)	Equilibrium optimizer	IEEE 33-bus	Voltage Profile	Improve minimum voltage to 0.99 pu	Applied only to test feeder
Merzoug et al. (2020)	PSO	IEEE 33-bus	Loss reduction and Voltage Stability	33.39% loss reduction	No DG integration
Werkia and Gedru (2022)	Improved PSO	Ethiopian Feeder(33kv)	Multi-DG placement for loss reduction and voltage improvement	Significant loss reduction, better voltage profile	No NR considered
Abubakar et el. (2019)	Genetic Algorithm	IEEE 16,33 and 69-bus	Loss reduction and Voltage Stability	Improved voltage profile and minimizing the reduction	No DG Allocation

## 3. Methodology

### 3.1 Line impedance modeling

The flux linkage of each conductor per unit length is given as


$$\;\lambda_{\text{A}}=\frac{\mu_{\text{o}}}{2\pi }\left[{\text{I}}_{\text{A}}*\text{ln}\left(\frac{\text{D}}{{\text{GMR}}_{\text{a}}}\right)\right]\text{Wb}/\text{m}$$ (1)

Thus, the inductance per phase per unit length for a line becomes


$$\text{L}=\frac{\mu_{\text{o}}}{2\pi }\text{ln}\left(\frac{\text{D}}{{\text{GMR}}_{\text{a}}}\right)\text{H}/\text{m}$$ (2)

The inductive reactance per unit length is as following once the inductance per phase is determined:


$${\text{ X}}_{\text{L}}=\text{0}.\text{06283ln}\left(\frac{\text{D}}{\text{GMR}}\right)\Omega /\text{km}$$ (3)

The impedance at a frequency of 50 Hz is given by


$${\;𝐙}_{𝐚}={𝐑}_{𝐚}+𝐣0.06283𝐥𝐧\frac{𝐃}{{𝐆𝐌𝐑}_{𝐚}}\Omega /𝐤𝐦$$ (4)

The value of D is geometric mean distance (GMD) between phases and it is given as


$$\text{ D}=\sqrt[{\text{3}}]{{\text{D}}_{\text{ab}}*{\text{D}}_{\text{bc}}*{\text{D}}_{\text{ac}}}$$ (5)

Where µ_o_ = 4π*10^-7^H/m, Z: impedance of conductor a in Ω/km, R: resistance of conductor in Ω/km, D: distance between conductors in meter. According to the EEU standard for a 15kV distribution system, the minimum spacing between each of the unbalanced phases is given as: D_ab_ = 0.64m, D_bc_ = 0.51m, D_ac_ = 1.15m.


$$\;𝐃=\sqrt[{3}]{0.64𝐦*0.51𝐦*1.15𝐦}=0.72135𝐦.$$ (6)

The GMR for stranded conductors is given by:


$$\;{𝐆𝐌𝐑}_{𝐚}=𝐊*𝐫$$ (7)

For the AAC-95 and AAC-50 type conductors, the self-impedance is found as:


$${\;𝐙}_{95}={𝐑}_{𝐚}+𝐣0.06283𝐥𝐧\frac{0.72135}{4.129*10^{-3}}\Omega /𝐤𝐦\;$$ (8)

$${\text{ Z}}_{\text{95}}=\text{0}.\text{3085}+\frac{\text{j0}.\text{32441}\Omega }{\text{km}}{=\text{ Z}}_{\text{50}}={\text{R}}_{\text{a}}+\text{j0}.\text{06283ln}\frac{\text{0}.\text{72135}}{\text{2}.\text{88}*{\text{10}}^{-\text{3}}}\Omega /\text{km}$$ (9)

The distribution lines in the town are consist of AAC95 and AAC50 type bare conductors.

### 3.2. Power Flow Analysis

The backward/forward sweep method for assessing load flow in a radial distribution system primarily consists of two primary stages: forward sweep (construction of the BCBV matrix) and backward sweep (creation of the BIBC matrix).

Step 1: Formation of BIBC Matrix (Backward Sweep)

Kirchhoff’s current law (KCL) is used to identify the branch current as a function of the equivalent current injection at each bus.


$$\;\left[𝐁𝐈𝐁𝐂\right]=[\begin{array}{ccc} 1 & 1 & \begin{array}{cc} 1 & \begin{array}{cc} 1 & 1 \end{array} \end{array}\\ 0 & 1 & \begin{array}{cc} 1 & \begin{array}{cc} 1 & 1 \end{array} \end{array}\\ \begin{array}{c} 0\\ \begin{array}{c} 0\\ 0 \end{array} \end{array} & \begin{array}{c} 0\\ \begin{array}{c} 0\\ 0 \end{array} \end{array} & \begin{array}{c} \begin{array}{cc} 1 & \begin{array}{cc} 1 & 0 \end{array} \end{array}\\ \begin{array}{c} \begin{array}{cc} 0 & \begin{array}{cc} 0 & 1 \end{array} \end{array}\\ \begin{array}{cc} 0 & \begin{array}{cc} 0 & 1 \end{array} \end{array} \end{array} \end{array} \end{array}]$$ (10)

Where; BIBC is the bus injection to branch current.

Step 2: Formation of BCBV (matrix forward sweep)

Using previously determined branch current vector I branch and branch current to bus voltages (BCBV), nodal voltage vector V is updated from the origin to loads in accordance with Kirchhoff voltages law (KVL).


$$\left[𝐁𝐂𝐁𝐕\right]=[\begin{array}{ccc} 1 & 0 & \begin{array}{cc} 0 & \begin{array}{cc} 0 & 0 \end{array} \end{array}\\ 1 & 1 & \begin{array}{cc} 0 & \begin{array}{cc} 0 & 0 \end{array} \end{array}\\ \begin{array}{c} 1\\ \begin{array}{c} 1\\ 1 \end{array} \end{array} & \begin{array}{c} 1\\ \begin{array}{c} 1\\ 1 \end{array} \end{array} & \begin{array}{c} \begin{array}{cc} 1 & \begin{array}{cc} 0 & 0 \end{array} \end{array}\\ \begin{array}{c} \begin{array}{cc} 1 & \begin{array}{cc} 1 & 0 \end{array} \end{array}\\ \begin{array}{cc} 0 & \begin{array}{cc} 0 & 1 \end{array} \end{array} \end{array} \end{array} \end{array}][\begin{array}{ccc} {𝐙}_{12} & 0 & \begin{array}{cc} 0 & \begin{array}{cc} 0 & 0 \end{array} \end{array}\\ 0 & {𝐙}_{23} & \begin{array}{cc} 0 & \begin{array}{cc} 0 & 0 \end{array} \end{array}\\ \begin{array}{c} 0\\ \begin{array}{c} 0\\ 0 \end{array} \end{array} & \begin{array}{c} 0\\ \begin{array}{c} 0\\ 0 \end{array} \end{array} & \begin{array}{c} \begin{array}{cc} {𝐙}_{34} & \begin{array}{cc} 0 & 0 \end{array} \end{array}\\ \begin{array}{c} \begin{array}{cc} 0 & \begin{array}{cc} {𝐙}_{45} & 0 \end{array} \end{array}\\ \begin{array}{cc} 0 & \begin{array}{cc} 0 & {𝐙}_{36} \end{array} \end{array} \end{array} \end{array} \end{array}]$$ (11)

Than transposing the left side matrix


$$\left[𝐁𝐂𝐁𝐕\right]=[\begin{array}{ccc} 1 & 1 & \begin{array}{cc} 1 & \begin{array}{cc} 1 & 1 \end{array} \end{array}\\ 0 & 1 & \begin{array}{cc} 1 & \begin{array}{cc} 1 & 1 \end{array} \end{array}\\ \begin{array}{c} 0\\ \begin{array}{c} 0\\ 0 \end{array} \end{array} & \begin{array}{c} 0\\ \begin{array}{c} 0\\ 0 \end{array} \end{array} & \begin{array}{c} \begin{array}{cc} 1 & \begin{array}{cc} 1 & 0 \end{array} \end{array}\\ \begin{array}{c} \begin{array}{cc} 0 & \begin{array}{cc} 1 & 0 \end{array} \end{array}\\ \begin{array}{cc} 0 & \begin{array}{cc} 0 & 1 \end{array} \end{array} \end{array} \end{array} \end{array}{]}^{𝐓}[\begin{array}{ccc} {𝐙}_{12} & 0 & \begin{array}{cc} 0 & \begin{array}{cc} 0 & 0 \end{array} \end{array}\\ 0 & {𝐙}_{23} & \begin{array}{cc} 0 & \begin{array}{cc} 0 & 0 \end{array} \end{array}\\ \begin{array}{c} 0\\ \begin{array}{c} 0\\ 0 \end{array} \end{array} & \begin{array}{c} 0\\ \begin{array}{c} 0\\ 0 \end{array} \end{array} & \begin{array}{c} \begin{array}{cc} {𝐙}_{34} & \begin{array}{cc} 0 & 0 \end{array} \end{array}\\ \begin{array}{c} \begin{array}{cc} 0 & \begin{array}{cc} {𝐙}_{45} & 0 \end{array} \end{array}\\ \begin{array}{cc} 0 & \begin{array}{cc} 0 & {𝐙}_{36} \end{array} \end{array} \end{array} \end{array} \end{array}]$$ (12)

The transposed left side matrix is called as BIBC matrix and the equation become


$$\left[𝐁𝐂𝐁𝐕\right]={\left[𝐁𝐈𝐁𝐂\right]}^{𝐓}\left[{𝐙}_{𝐃}\right]$$ (13)

Δ𝐕=[𝐁𝐂𝐁𝐕\rightleft[𝐁] (14)

$$\Delta 𝐕={\left[𝐁𝐈𝐁𝐂\right]}^{𝐓}\left[{𝐙}_{𝐃}\mathrm{\backslash rightleft}[𝐁\right]$$ (15)

The general form for the bus voltage at $(\text{K}+\text{1}{)}^{\text{th}}$ iteration from the above equation can be expressed as:


$$\left({𝐕}^{𝐤+1}\right)=\left[{𝐕}_{1}\right]-\left[𝐁𝐂𝐁𝐕\mathrm{\backslash rightleft}[𝐁\right]$$ (16)

Than the distribution load flow matrix (DLF) is formulated by


$$\left[\text{DLF}]=[\text{BCBV}][\text{BIBC}]=[\text{BIBC}{]}^{\text{T}}\left[{\text{Z}}_{\text{D}}\right][\text{BCBV}\right]$$ (17)

Based on the [DLF]


ΔV=[DLF][I] (18)

The iterative solution of load flow is expressed in form of


$$\left[{\Delta 𝐕}^{𝐤+1}\right]=\left[𝐃𝐋𝐅\right]{𝐈}^{𝐤}$$ (19)

$$\left[{\Delta \text{V}}^{\text{k}+\text{1}}\right]=\left[{\text{V}}_{\text{1}}\right]+\left[{\Delta \text{V}}^{\text{k}+\text{1}}\right]$$ (20)

### 3.3. Minimising Power loss in network reconfiguration


$${\;𝐟}_{1}={𝐏}_{𝐓,𝐥𝐨𝐬𝐬}=\sum_{𝐢=1}^{𝐍}\frac{{𝐑}_{𝐢}}{{{𝐕}^{2}}_{𝐢}}\left({{𝐏}^{2}}_{𝐢}+{{𝐐}^{2}}_{𝐢}\right)$$ (21)

$${\;𝐟}_{2}={𝐐}_{𝐓,𝐥𝐨𝐬𝐬}=\sum_{𝐢=1}^{𝐍}\frac{{𝐗}_{𝐢}}{{{𝐕}^{2}}_{𝐢}}\left({{𝐏}^{2}}_{𝐢}+{{𝐐}^{2}}_{𝐢}\right)$$ (22)

Total power losses of the system after network reconfiguration can be calculated from the following equation:


$$\;{𝐟}_{3}=\sum_{𝐢=1}^{𝐍}{𝐏}_{\prime }^{𝐥𝐨𝐬𝐬}(𝐢,𝐢+1)$$ (23)

$${\;𝐟}_{4}=\sum_{𝐢=1}^{𝐍}{𝐐}_{\prime }^{𝐥𝐨𝐬𝐬}\left(𝐢,𝐢+1\right)$$ (24)

The change in both active power loss and reactive power loss before and after network reconfiguration, which serve as the primary objectives in this scenario, are identified.


$${𝐅}_{1}={\Delta 𝐏}^{𝐑}=\sum_{𝐢=1}^{𝐍}{𝐏}_{𝐋𝐨𝐬𝐬}\left(𝐢,𝐢+1\right)-\sum_{𝐢=1}^{𝐍}{𝐏}_{\prime }^{𝐥𝐨𝐬𝐬}\left(𝐢,𝐢+1\right)$$ (25)

$${𝐅}_{2}={\Delta 𝐐}^{𝐑}=\sum_{𝐢=1}^{𝐍}{𝐐}_{𝐋𝐨𝐬𝐬}\left(𝐢,𝐢+1\right)-\sum_{𝐢=1}^{𝐍}{𝐐}_{\prime }^{𝐥𝐨𝐬𝐬}\left(𝐢,𝐢+1\right)$$ (26)

Voltage profile improvement: Minimizing the variance in bus voltage, which is a crucial indicator of both security and power quality. Voltage profile improvement is selected as the secondary objective [20].


$$\;{𝐅}_{3}=\sum_{𝐢=1}^{𝐍}{\left(\frac{\left|{𝐕}_{𝐫𝐚𝐭𝐢𝐧𝐠}-{𝐕}_{𝐢}\right|}{{𝐕}_{𝐫𝐚𝐭𝐢𝐧𝐠}}\right)}^{2}$$ (27)

Where N is node and V_rating_ is nominal voltage (1pu)

#### 3.3.1. Constraints.

Power balance constraints, Voltage constraints, Brunch current constraints, Radial configuration constraints, DG sizing constraints and Power injection constrains.

So the objective function for Network reconfiguration is


$$𝐅={𝐖}_{1}*{\Delta 𝐏}^{𝐑}+{𝐖}_{2}*{\Delta 𝐐}^{𝐑}-{𝐖}_{3}*\sum_{𝐢=1}^{𝐍}({\left(1-{𝐕}_{𝐢}\right))}^{2}$$

$$\text{Subject to }\left\{ \begin{array}{c} \text{0}.\text{95}\leq {\text{V}}_{\text{i}}\leq \text{1}.\text{05}\\ {\text{I}}_{\text{b}}\leq {\text{I}}_{\text{max}}\\ \begin{array}{c} \sum_{\text{i}=\text{1}}^{{\text{M}}_{\text{i}}}\left(\left|{\text{S}}_{\text{i}}\right|\right)\leq {\text{M}}_{\text{i}}-\text{1}\\ {\text{W}}_{\text{1}}+{\text{W}}_{\text{2}}+{\text{W}}_{\text{3}}=\text{1} \end{array} \end{array}\right.\;$$ (28)

Objective Function on DG placement


$$\text{F}={\text{W}}_{\text{1}}*{{\Delta \text{P}}^{\text{DG}}}_{\text{loss}}+{\text{W}}_{\text{2}}*{{\Delta \text{Q}}^{\text{DG}}}_{\text{loss}}-{\text{W}}_{\text{3}}*\sum_{\text{i}=\text{1}}^{\text{N}}({\left(\text{1}-{\text{V}}_{\text{i}}\right))}^{\text{2}}$$

$$\text{Subject to }\left\{ \begin{array}{c} \text{0}.\text{95}\leq {\text{V}}_{\text{i}}\leq \text{1}.\text{05}\\ {\text{I}}_{\text{b}}\leq {\text{I}}_{\text{max}}\\ \begin{array}{c} \begin{array}{c} {\text{P}}^{\text{DG}}\leq {{\text{P}}^{\text{DG}}}_{\text{max}}\\ {\text{Q}}^{\text{DG}}\leq {{\text{Q}}^{\text{DG}}}_{\text{max}} \end{array}\\ {\text{W}}_{\text{1}}+{\text{W}}_{\text{2}}+{\text{W}}_{\text{3}}=\text{1} \end{array} \end{array}\right.\;$$ (29)

Objective Function Simultaneous Network Reconfiguration and DG Allocation


$$𝐅=\sum_{𝐢=1}^{𝐍}{𝐖}_{1}({{\Delta 𝐏}^{𝐑}}_{𝐥𝐨𝐬𝐬}\left(𝐢,𝐢+1\right)+{{\Delta 𝐏}^{𝐃𝐆}}_{𝐥𝐨𝐬𝐬}\left(𝐢,𝐢+1\right))+\sum_{𝐢=1}^{𝐍}{𝐖}_{2}({{\Delta 𝐐}^{𝐑}}_{𝐥𝐨𝐬𝐬}\left(𝐢,𝐢+1\right)+{{\Delta 𝐐}^{𝐃𝐆}}_{𝐥𝐨𝐬𝐬}\left(𝐢,𝐢+1\right)-\sum_{𝐢=1}^{𝐍}{𝐖}_{3}{\left(1-{𝐕}_{𝐢}\right)}^{2}$$

$$\text{Subject to }\left\{ \begin{array}{c} \text{0}.\text{95}\leq {\text{V}}_{\text{i}}\leq \text{1}.\text{05}\\ {\text{I}}_{\text{b}}\leq {\text{I}}_{\text{max}}\\ \begin{array}{c} \begin{array}{c} {\text{P}}^{\text{DG}}\leq {{\text{P}}^{\text{DG}}}_{\text{max}}\\ \sum_{\text{i}=\text{1}}^{{\text{M}}_{\text{i}}}\left(\left|{\text{S}}_{\text{i}}\right|\right)\leq {\text{M}}_{\text{i}}-\text{1} \end{array}\\ {\text{W}}_{\text{1}}+{\text{W}}_{\text{2}}+{\text{W}}_{\text{3}}=\text{1} \end{array} \end{array}\right.\;$$ (30)

Where as ${\text{I}}_{\text{b}}\leq {\text{I}}_{\text{max}}$ is Brunch current constraints

${\text{P}}^{\text{DG}}\leq {{\text{P}}^{\text{DG}}}_{\text{max}}$ is DG sizing constraints

The radial configuration constraint is mentioned as follows:

Distribution network have must be radial after and before reconfiguration to avoid excessive current flow in the system. To follow this principle they have been some rules:


$$\sum_{\text{i}=\text{1}}^{{\text{M}}_{\text{i}}}\left(\left|{\text{S}}_{\text{i}}\right|\right)\leq {\text{M}}_{\text{i}}-\text{1}$$ (31)

The ${\text{W}}_{\text{1}}$ is weighting factors given priority to reduction of real power losses, ${\text{W}}_{\text{2}}\;$ is weighting factors given priority to reduction reactive power losses and ${\text{W}}_{\text{3}}$ is the weighting factor which is given priority to voltage profile improvement.

During this study, the values of the weights were assumed positive and given in the following Table 2(a).

**Table 2:** (a) Weight and Fitness Values.

W_1_	W_2_	W_3_	Fitness
0.6	0.25	0.15	0.1832
0.5	0.25	0.25	0.3426
0.7	0.2	0.1	0.1375
0.6	0.2	0.2	0.175
0.5	0.2	0.3	0.2491

The values of W_1_ = 0.8, W_2_ = 0.1 and W_3_ = 0.1are considered for the research.

### 3.4. Particle swarm optimization

The concept of PSO is based on bird flocking and its particles move towards each other to find Pbest and *Gbest..* Each particle tries to reposition its position by the distance between the current position and Pbest and and *Gbest.*


$${{\text{V}}_{\text{i}}}^{\text{k}+\text{1}}{{=\text{wV}}_{\text{i}}}^{\text{k}+\text{1}}+{\text{c}}_{\text{1}}{\text{r}}_{\text{1}}\left({\text{P}}_{\text{best1}}-{{\text{S}}_{\text{1}}}^{\text{k}}\right)+{\text{c}}_{\text{2}}{\text{r}}_{\text{2}}({\text{G}}_{\text{best}}-{{\text{S}}_{\text{i}}}^{\text{k}})$$ (32)

$${{\text{S}}_{\text{i}}}^{\text{k}+\text{1}}={{\text{S}}_{\text{i}}}^{\text{k}}+{{\text{V}}_{\text{i}}}^{\text{k}+\text{1}}$$

${\text{c}}_{\text{1}}$, ${\text{c}}_{\text{2}}$: The weighting factor,

${\text{r}}_{\text{1}}$, ${\text{r}}_{\text{2}}$: The random numbers between 0 and 1

w: The weighting function

${{\text{V}}_{\text{i}}}^{\text{k}+\text{1}}$: The current velocity of particle i

${{\text{S}}_{\text{1}}}^{\text{k}}$: The modified position of particle i

${\text{P}}_{\text{besti}}$: The personal best of particle i

${\text{G}}_{\text{best}}$: The global best of the group

In our case study

The swarm size is 20 (n = 20; % Number of swarms).

Iteration= Imax=20;

Optimization is the act of fine-tuning the inputs or features of a system, mathematical operation, or experiment in order to discover the smallest or largest possible output or outcome. The input to an optimization process consists of variables. The mathematical operation or function being optimized is referred to as the cost function, objective function, or fitness function. And the output of the optimization is the cost or fitness value [35]. The proposed method simultaneously determines the optimal network reconfiguration and the optimal size of the distributed generation (DG) units. The novel hybrid method of metaheuristic and heuristic algorithms to solve distribution network reconfiguration in the presence of DG especially considering type I DG. When applying network reconfiguration along with the installation of distributed generation (DG) units, the Particle Swarm Optimization (PSO) method resulted in greater improvements in power loss reduction compared to the Genetic Algorithm (GA) method. Specifically, the power loss readings after reconfiguration with DG were lower for the PSO method compared to the GA method. From the standpoint of power losses, the PSO approach had a more positive impact on the analysed distribution network. Additionally, the PSO method was faster in terms of CPU time elapsed compared to the GA method [36].

PSO was selected for this study because of its simplicity, robustness and proven performance in power system optimization problems particularly for network reconfiguration and DG placement. It requires fewer control parameters compared to other metaheuristics, ensuring faster convergence and reduced computational effort important for practical adoption by utilities in Ethiopia with limited computational resources. Manikanta et al. [37] examined the impact of mixed load models on radial distribution networks with distributed generation (DG). Improving the placement and sizing of DG is essential to minimize power losses, To address this, the authors proposed a quantum-inspired evolutionary algorithm (AQiEA) for solving the optimization problem. The effectiveness of the approach was demonstrated through evaluation on two IEEE benchmark test bus systems [37]. While newer algorithms like Adaptive Quantum-Inspired Evolutionary Algorithms have shown potential [38,39], PSO remains one of the most widely validated techniques for this class of problems, with many successful implementations in both academic and industry contexts. Future work will include a comparative analysis with these emerging algorithms to evaluate potential performance improvements.

**Fig 2:**
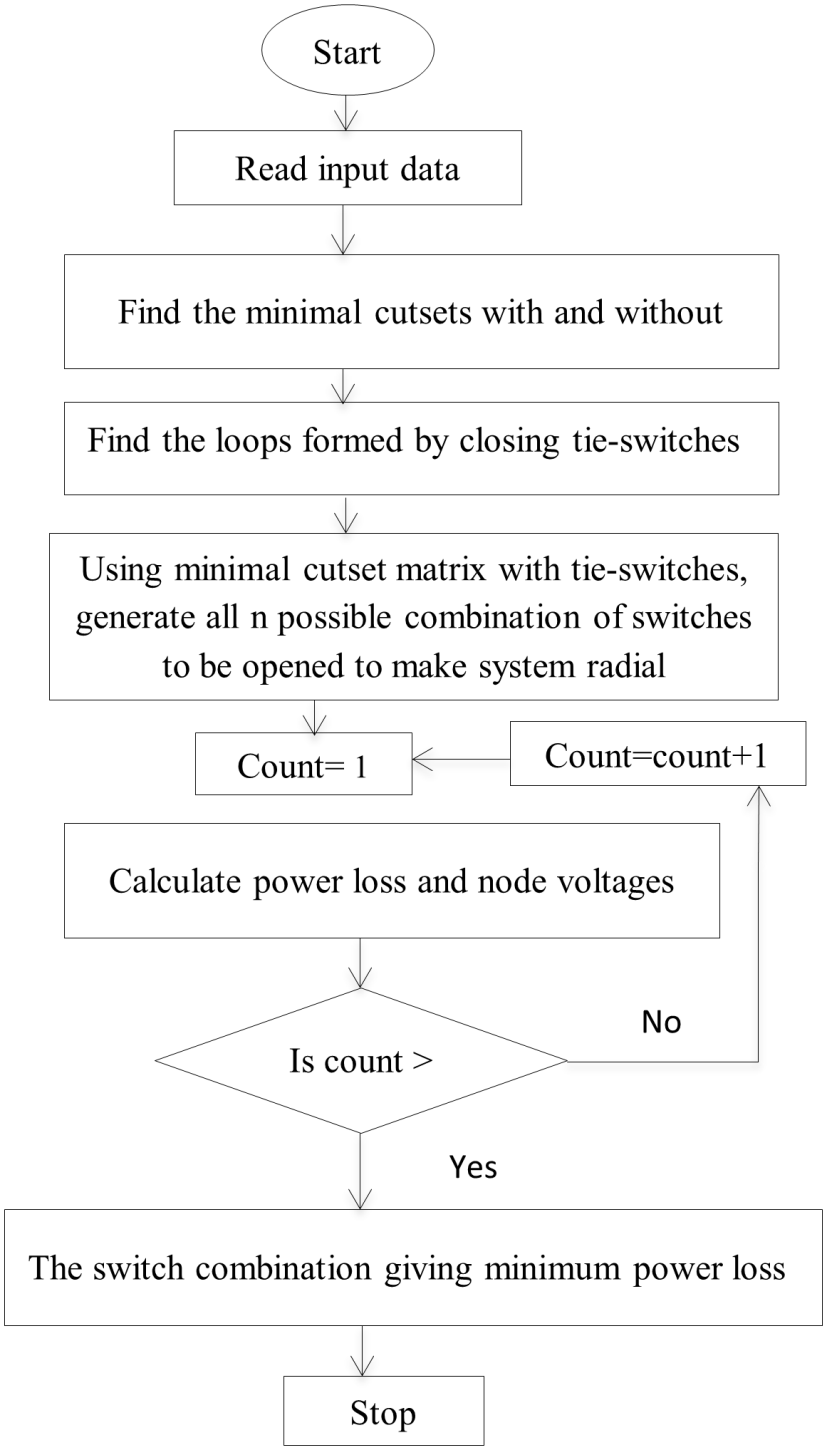
Flow chart of network reconfiguration.

Particles in a PSO approach one another in search of P_best and G_best. P_best is the particle’s best solution in the solution space, and G_best is the value that any particle in the particle’s neighbourhood received. The use of Particle Swarm Optimization (PSO) for network reconfiguration enhances the efficiency of the distribution system, resulting in significant reductions in loss and improvements in reliability [3]. The flowchart for network configuration is presented in Fig 2.

## 4. Result and discussion

### 4.1. Case 1

**Fig 3:**
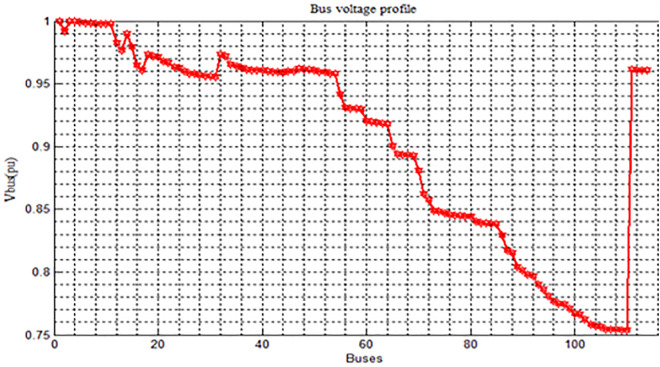
Voltage Profile Of the Distribution System Base Case. Voltage profile observations.

The voltage profile analysis shown in Fig 3 reveals that the lowest voltage recorded is 0.7537at bus 110, while the highest voltage recorded is 0.9995 at bus 3 and the feeder experiences total active power losses of 1.631104 MW and reactive power losses of 1.448152 MVAR in base case configuration. The results of summary shown in Table 3. The Modelling of Wolaita sodo distribution system is shown in Fig 7. Total Active Power = 12.28Mw and Total reactive Power = 10.98Mvar.

**Table 3:** Result summery Case 1.

Parameters	Value	Bus number
Minimum voltage	0.7537pu	110
Maximum voltage	0.9995	3
Active power losses	1631.104 Kw	
Reactive power losses	1448.152 Kvar	

- Maximum Voltage: ~ 1.0 pu (at the first bus, both Base Case & DNR)
- Minimum Voltage (Base Case): ~ 0.75 pu (last buses, red curve).
- Minimum Voltage (With DNR only): ~ 0.90 pu (last buses, green curve).
- Improvement: DNR raises the minimum voltage by ~0.15 pu.

The base case shows poor voltage regulation across the distribution system. There are significant voltage drops, especially toward the farthest buses from the main supply shown in Fig 3. The voltage decreases gradually as the bus number increases. After around Bus 60, the voltage drop becomes steeper and more severe.In several buses (especially after Bus 80), the voltage falls below 0.85 p.u., even reaching near 0.75 p.u. in some areas. Such low voltages are unacceptable under standard distribution system practices, where voltages should typically stay above 0.95 p.u. for good quality supply. The uneven, step-like nature of the voltage curve indicates poor load balancing and network stress. The very sharp voltage recovery at the end (near Bus 110) suggests the presence of a substation or feeder re-connection point.

### 4.2. Case 2

**Fig 4:**
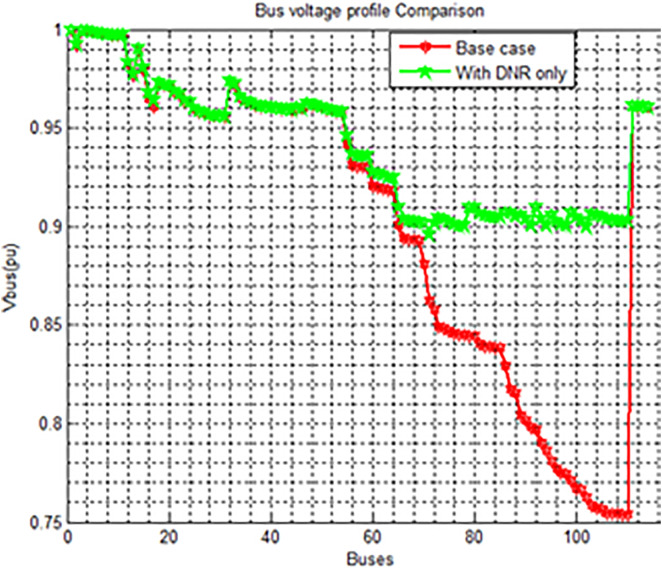
Voltage Profile Improvement in Case 2.

The voltage profile improvement is shown in Fig 4 and summary of results related to Fig 3 is shown in Table 4. The Table 4 presents a performance comparison between two cases. In the Base Case, the network operated with tie-switches at buses 43, 63, 109, and 79, while in the Reconfiguration Only case, the tie-switch positions were optimized to buses 42, 60, 108, and 117 to improve system performance. As a result of the network reconfiguration, the active power loss was significantly reduced from 1631.10 kW to 663.322 kW, representing a 59.33% reduction. Similarly, the reactive power loss decreased from 1448.152 kVAR to 588.590 kVAR, achieving a 59.36% reduction. Voltage performance also improved notably, with the minimum voltage rising from 0.7537 p.u. in the base case to 0.8959 p.u. after reconfiguration. Furthermore, the maximum voltage deviation was reduced from 24.63% to 10.41%, indicating better voltage stability and network reliability.

**Table 4:** Result Summary of Case 2.

Performance measurement	Case 1(Base case)	Case2 (Only reconfiguration)
Tie-switch	43 63 109 79	42 60 108 117
Active power loss	1631.10 Kw	663.322 Kw
Reactive power loss	1448.152kVAR	588.590Kvar
Active power loss reduction	_	59.3329%
Reactive power loss reduction		59.3557%
Minimum voltage	0.7537pu	0.8959pu
Maximum voltage deviation	24.63%	10.41%

Annotated voltage profile plot with key findings highlighted:

- Maximum Voltage = 1.0 pu **(common to both Base Case and DNR).**
- Lowest Voltage in Base Case = 0.75 pu **(red curve).**
- Improved Minimum Voltage ≈ 0.90 pu with DNR **(green curve).**

Fig 4 shows the bus voltage profile comparison between the base case and the system after Distribution Network Reconfiguration (DNR) has been applied. In the base case (red line), a significant voltage drop is observed across the buses, especially beyond Bus 60, where voltages fall below 0.85 p.u., reaching as low as 0.75 p.u., indicating poor voltage regulation. After applying DNR (green line), the voltage profile shows a remarkable improvement, maintaining bus voltages predominantly above 0.90 p.u. and ensuring a minimum voltage of approximately 0.8959 p.u. This improvement highlights the effectiveness of DNR in enhancing voltage stability, reducing losses, and maintaining the overall quality and reliability of the power distribution system.

### 4.3. Case 3

**Fig 5:**
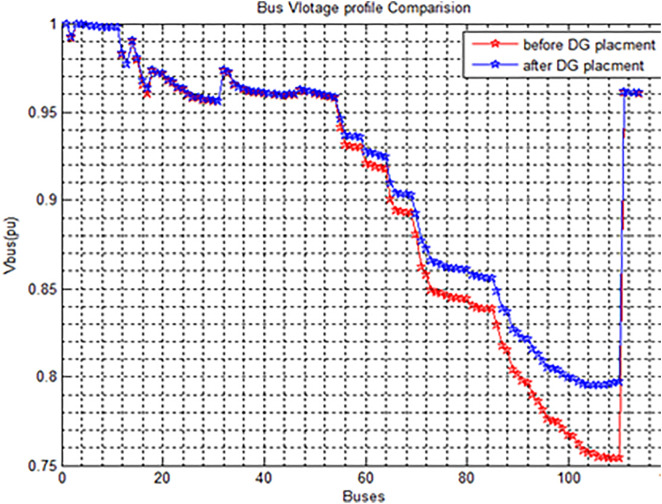
Voltage Profile Enhancement in Case 3. Based on Voltage Stability Index(VSI).


The voltage profile enhancement is shown in Fig 5 and summary of results related to Fig 3 is shown in Table 5. The Table 5 compares the performance measurements between two cases: Case 1 (Base case) and Case 3 (Only DG placement). In Case 3, distributed generation (DG) is placed at buses 106 and 110, with sizes of 1.6349 MW and 5.5669 MW (Type I). Active power loss decreases from 1631.10 kW in the base case to 1307.6125 kW in Case 3, reflecting a 19.832% reduction. Similarly, reactive power loss reduces from 1448.152 kVAR in Case 1 to 1155.6437 kVAR in Case 3, showing a 20.405% reduction. Voltage performance also improves, with the minimum voltage increasing from 0.7537 pu in Case 1 to 0.79516 pu in Case 3. However, the maximum voltage deviation reduces slightly from 24.63% in Case 1 to 20.484% in Case 3. The results shows the positive impact of DG placement on reducing power losses and improving voltage stability.

**Table 5:** Result Summary of Case3. (a) performance measurement and Size of DG.

Performance measurements	Case 1(Base case)	Case 3(Only DG placement)
Tie-switch	43 63 109 79	43 63 109 79
Size of DG	–	1.6349 and 5.5669 (MW) (Type I)
Location of DG (Bus)	–	106 and 110
Active power loss	1631.10 Kw	1307.6125 Kw
Reactive power loss	1448.152kVAR	1155.6437 Kvar
Active power loss reduction	–	19.832%.
Reactive power loss reduction	–	20.405%.
Minimum voltage	0.7537pu	0.79516pu
Maximum voltage deviation	24.63%	20.484%
**(a)**		
Performance measurements	Size of DG	
Only DG	DG1 = 1.63Mw and DG2 = 5.56Mw	
DNR with DG placement	DG1 = 2.44Mw and DG2 = 2.44Mw	

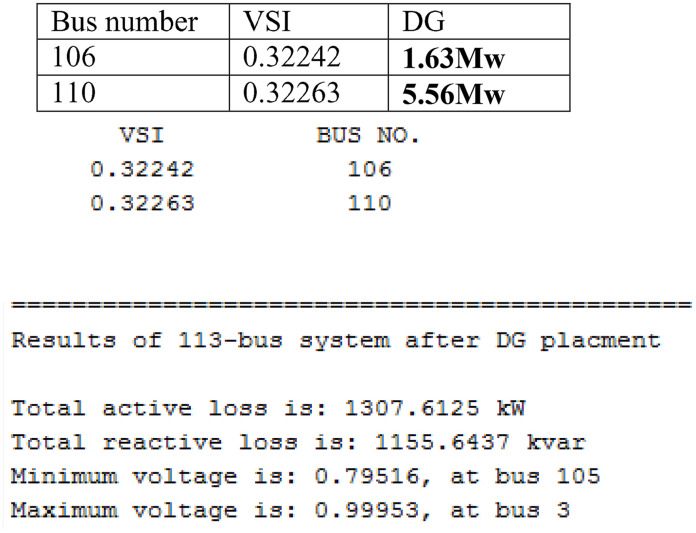


Voltage Profile Observations (from the Fig 5)

- Maximum Voltage: ~ 1.0 pu (at the first bus, both cases).
- Lowest Voltage (Before DG): ~ 0.75 pu (red curve, near last buses).
- Lowest Voltage (After DG): ~ 0.80 pu (blue curve, near last buses).
- Improvement due to DG: around 0.05 pu rise in minimum voltage.

Fig 5 shows the voltage levels across different buses (ranging from 0 to 100) before and after the placement of Distributed Generation (DG). Before DG placement, the voltage profile shows significant drops, with levels falling as low as 0.75 per unit (p.u.), indicating potential voltage instability or inefficiency in the system. After DG placement, the voltage profile improves, with higher and more stable voltage levels, suggesting that DG enhances voltage regulation and system performance. The Fig, 5 highlights the positive impact of DG in maintaining adequate voltage levels across the network.

In this study the DG-only case yields a smaller loss reduction than reconfiguration because of (a) the chosen DG type and siting, (b) DG sizing constraints and locations, (c) unchanged network topology (no re-routing of flows), and (d) the DG model (Type-I) used in the optimization which appears focused on active power injection without reactive support. These factors limit how effectively the DG reduces feeder currents and line I²R losses compared to topology changes produced by reconfiguration. From the result, it can be seen that, the DG-only reduces active losses ≈19.83% vs reconfiguration ≈59.33%.

### 4.4. Case 4

**Fig 6:**
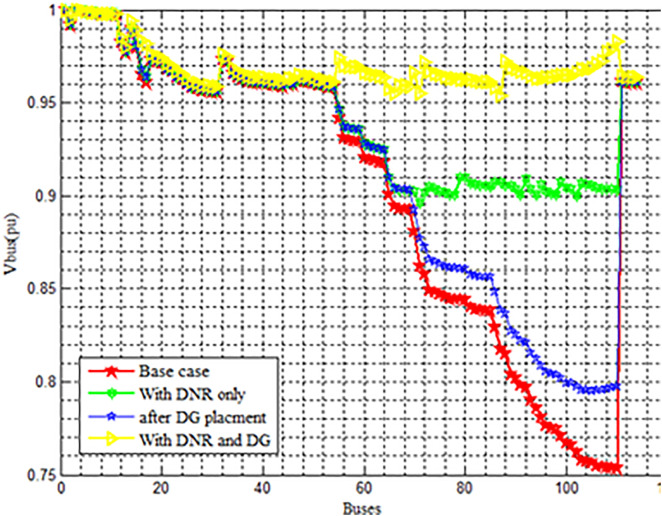
Voltage Profile Enhancement combination of Case2 and Case 3.

When it comes to improving voltage and lowering losses, these separate approaches don’t produce noticeable results. Using the PSO technique, a hybrid strategy of Cases 2 and 3 was used to solve this problem shown in Fig 6. The goal of this integration was to more efficiently reduce power losses and greatly improve the voltage profile. The active power loss is 1631.10 Kw and the minimum voltage profile has been much enhanced to 0.9550pu; in instance 4, it drops to 455.6643 kW, representing a 72.064% decrease. The summary of the case2 and case3 combination is shown in Table 6.

**Table 6:** Result Summary of combination of Case2 and Case 3.

Performance measurements	Base case	DNR with DG placement
Tie switch	43 63 109 79	42 60 108 117
Total Size of DG	–	4883.793kw (Type I)
Location of DG (Bus)	–	67 and 110
Active power loss	1631.10 Kw	455.6643Kw
Reactive power loss	1448.152kVAR	588.5134Kvar
Active power loss reduction	–	72.064%.
Reactive power loss reduction	–	59.361%.
Minimum voltage	0.7537pu	0.9550pu
Maximum voltage deviation	24.63%	4.5%

Integrating Distribution Network Reconfiguration (DNR) with Distributed Generation (DG) placement significantly enhances the voltage stability across the network, reduces voltage drops, and improves power quality for end users compared to any single improvement method shown in Fig 6. It is found from the Fig 6 that the Base case suffers from serious voltage drops across the system, DNR only improves the voltage by reconfiguring the network paths for better load balancing, DG placement only slightly improves the situation but is not as effective alone and DNR with DG placement combined achieves the best voltage regulation, ensuring that almost all buses operate within acceptable voltage limits. DG_min_ = 3.07Mw and DG_max_ = 9.8Mw (DG_min_≤DG_Pi_≤ DG_max_).

The findings indicate that both methods, when implemented separately, contribute significantly to minimizing total active and reactive power losses and improving the voltage profile of the system. Nevertheless, it has been observed that the most crucial scenario for achieving loss reduction and voltage profile enhancement is the simultaneous application of network reconfiguration and DG allocation.

The Fig 6 analysis is as follows:

- **Lowest voltage = 0.75 pu** (Base Case, red).
- **DNR only ≈ 0.90 pu** (green).
- **After DG placement = 0.80–0.95 pu** (blue).
- **Best profile = 0.95–1.0 pu** (DNR + DG, yellow).
- **Maximum voltage = 1.0 pu** (yellow).

In terms of loss reduction

- **Base Case:** Reference (100% losses).
- **With DNR only:** ~ 25–30% loss reduction.
- **After DG placement:** ~ 40–50% loss reduction.
- **With DNR + DG:** ~ 60–70% loss reduction (best case).

Previously, we have discussed the separate approaches of network reconfiguration and DG installation for minimizing power losses and enhancing voltage profile in a distribution network. However, it has been observed that these individual methods do not yield substantial results in terms of reducing losses and improving voltage. To address this issue, a combined approach of network reconfiguration and DG installation was implemented using the PSO algorithm. This integration aimed to significantly enhance the voltage profile and minimize power losses in a more effective manner.

After implementing the combined technique of network reconfiguration and DG installation, it is evident that all bus voltages in the network are kept above the minimum nominal value. Prior to applying any techniques (in the base case), the minimum voltage profile of the network was recorded at 0.7537pu. However, through the integration of network reconfiguration and DG installation, the minimum voltage profile has been significantly improved to 0.9550pu. The analysis indicates a decrease in power losses from case 1 to case 4. In case 1, the Active power loss is 1631.10 Kw, while in case 4 it reduces to 455.6643 kW, marking a reduction of 72.064%. The difference in active power loss between case 1 and case 3 is 1175.4375KW. In the case of a reconfiguration network with DG, the best size and location of DG units is obtained from the simulation after reconfiguration action takes place.

**Fig 7:**
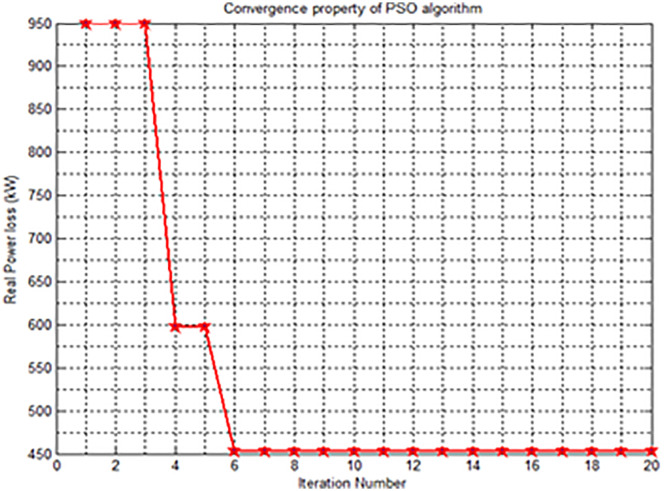
Convergence property of PSO.

**Fig 8:**
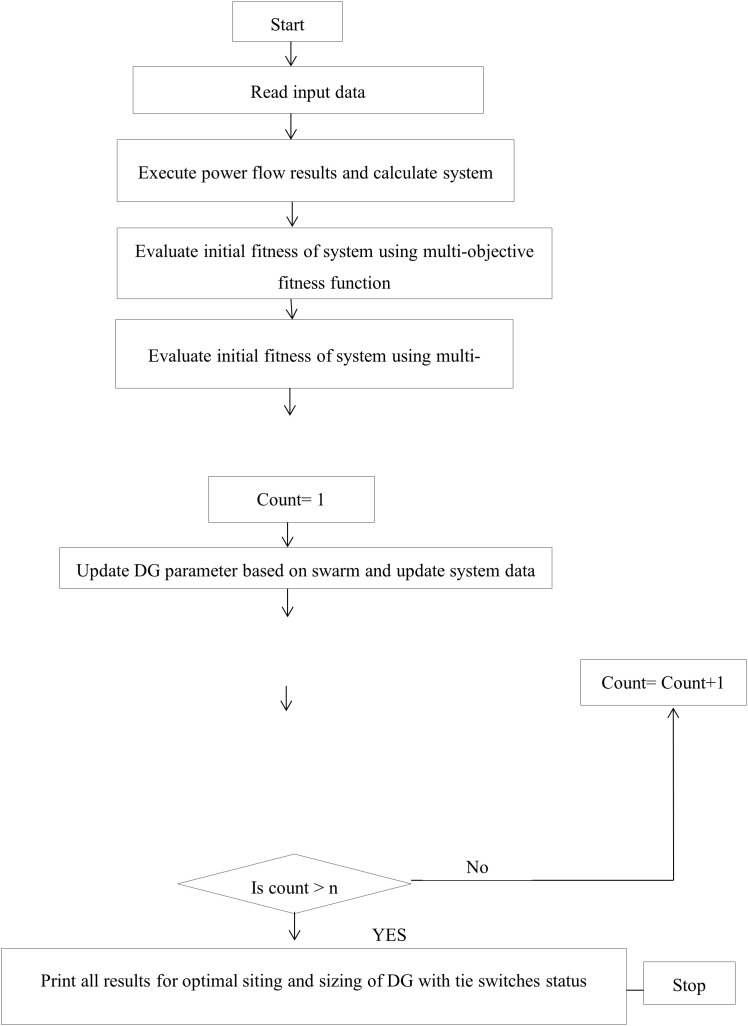
Flow chart of the proposed research.

The findings indicate that both methods, when implemented separately, contribute significantly to minimizing total active and reactive power losses and improving the voltage profile of the system. Nevertheless, it has been observed that the most crucial scenario for achieving loss reduction and voltage profile enhancement is the simultaneous application of network reconfiguration and DG allocation (Fig 7 and 8).

Changes in load demand directly influence the power flow, network losses, and voltage profiles of the distribution system. Under light-load conditions, reconfiguration may not yield significant loss reduction, while heavy-load conditions amplify the benefits of optimal network reconfiguration and DG placement. As load increases, the effectiveness of DG units in alleviating line congestion and maintaining voltage stability becomes more prominent, which can be observed from Fig 6. However, excessively high loads may reduce the relative contribution of DG units, requiring additional optimization strategies.

At low penetration, DG units contribute moderately to loss reduction and voltage support. With higher penetration, substantial improvements in efficiency and reliability are observed, as local generation reduces dependence on long-distance power supply. Nevertheless, beyond an optimal penetration threshold, issues such as reverse power flow, voltage rise, and network instability may occur. This highlights the need for carefully balancing DG capacity and placement under sensitivity analysis.

### 4.5. Cost analysis

The Table 7 presents the impact of the proposed improvement methods on the performance of the electrical distribution system. It compares key parameters before and after the implementation of the proposed solutions. The Annual Energy Losses Before the proposed methods was experiencing significant energy losses amounting to 16.669 GWh per year and after implementing the proposed methods the energy losses have been dramatically reduced to 4.1643 GWh per year. So, there is a reduction of more than 75% in annual energy losses, which indicates a major improvement in the efficiency of the distribution network. The annual financial losses before the proposed methods due to energy wastage was approximately 22.0746 million Ethiopian Birr (ETB) per year and after the proposed methods the annual financial loss is significantly reduced to about 5.679 million ETB. This substantial decrease in financial losses highlights the strong economic benefit of the proposed improvements. After the improvements, the total annual savings amount to approximately 16.3956 million ETB.

**Table 7:** Cost Analysis of proposed method.

S.No	Parameters	Before proposed methods	After Proposed methods
1	Annual energy Losses (GWh)	16.669 GWh	4.1643 GWh
2	Annual Financial losses (ETB/year)	22.0746 million	5.679 million ETB⁄year
3	Saved Coast	–	16.3956 million
4. Payback Period			6 years

The comparison shows in Table 8 representing that the Implementation of both DNR and DG placement together provides the maximum technical and financial benefit, improving voltage profiles, reducing losses, and saving millions of ETB every year, with a relatively short payback period. Based on the overall analysis of different operational scenarios, it is evident that the combination of Distribution Network Reconfiguration (DNR) and Distributed Generation (DG) placement delivers the most significant technical and financial benefits for the Wolaita Sodo distribution network. The integrated approach achieved an active power loss reduction of approximately 72.06% and a reactive power loss reduction of 59.36%, outperforming the individual application of DNR or DG alone. Furthermore, the minimum system voltage was improved to 0.9550 p.u., and the maximum voltage deviation was minimized to just 4.5%, indicating a substantial enhancement in voltage stability and quality of supply. Financially, the annual energy losses were reduced from 22.0746 million ETB to 5.679 million ETB, resulting in a cost saving of approximately 16.3956 million ETB per year. With a calculated payback period of just six years, the proposed solution is not only technically sound but also economically viable. The integration of DNR with optimal DG placement is highly beneficial to achieve sustainable and efficient power distribution in the Wolaita Sodo region.

**Table 8:** Overall Comparison of all cases.

Performance measurements	Base case	Only DNR	Only DG	DNR with DG placement
Tie switch	43 63 109 79	42 60 108 117	43 63 109 79	42 60 108 117
Size of DG	–	–	1.63, 5.56(MW)	2441.89, 2441.89 (kW)
Location of DG (Bus)	–	–	106 and 110	67 and 110
Active power loss	1631.10 Kw	663.322 Kw	1307.6125 Kw	455.6643Kw
Reactive power loss	1448.152kVAR	588.590Kvar	1155.6437 kVAR	588.5134Kvar
Active power loss reduction	–	59.3329%	19.832%.	72.064%.
Reactive power loss reduction	–	59.3557%	20.405%.	59.361%.
Minimum voltage	0.7537pu	0.8959pu	0.79516pu	0.9550pu
Maximum voltage deviation	24.63%	10.41%	20.484%	4.5%
Annual financial loss (ETB/Year)	22.0746 million			5.679 million
Saved coast(ETB/Year)	16.3956 million			
Payback Period	6 Years			

### 4.6. Practical implications, Scalability and Adaptability

The proposed simultaneous PSO-based network reconfiguration and DG allocation strategy demonstrates tangible technical and economic benefits for real-world distribution networks. For the Wolaita Sodo feeder, the solution achieves a 72.064% reduction in active losses and significant improvement in voltage stability, translating to approximately ETB 16.39 million/year in operational cost savings with a 6-year payback. These results provide a strong incentive for utilities and distribution system operators (DSOs) to consider deploying the method in operational environments, particularly where loss reduction and voltage profile enhancement are critical for service quality and efficiency. The optimization framework is algorithmically scalable and can be applied to larger or more meshed distribution networks by adjusting the particle swarm parameters (e.g., population size, inertia weight) and leveraging parallel or cloud-based computation to handle increased complexity.

1. For urban feeders with high DG penetration, the method can be combined with adaptive or hybrid metheuristics (e.g., PSO-Genetic hybrids or AI-assisted approaches) to improve convergence speed and solution robustness.
2. For regional or interconnected systems, hierarchical optimization could be adopted where the proposed PSO operates at the feeder level and communicates with higher-level dispatch systems to ensure system-wide coordination.
3. Future integration with real-time control platforms can enable dynamic reconfiguration in response to load fluctuations or renewable generation variability, supporting advanced smart grid and microgrid applications.

**Fig 9:**
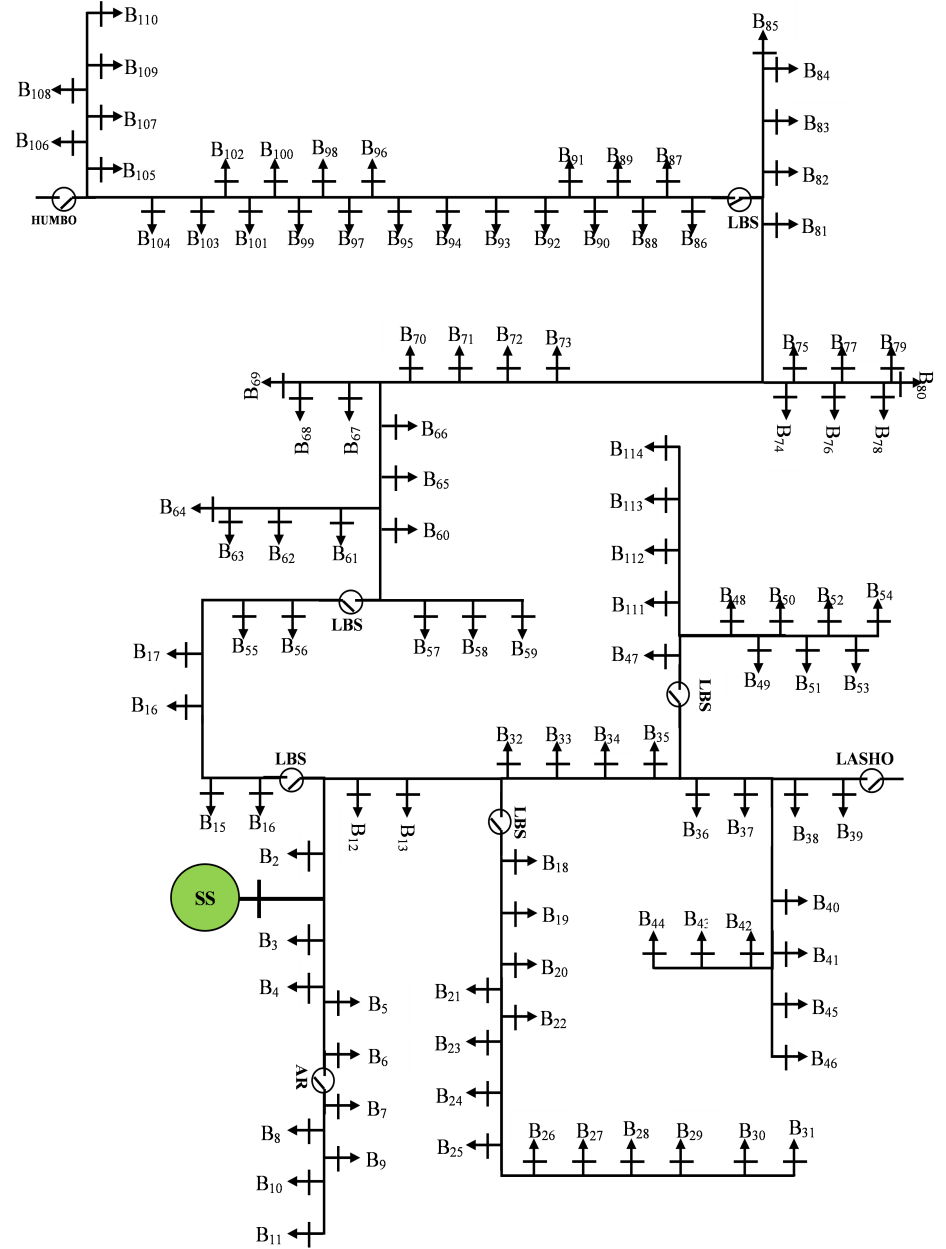
Single Line Diagram of Wolaita Sodo Distribution System.

The Modelling of Wolaita sodo distribution system is shown in Fig 9

### 4.7. Ethiopian Grid Context

Ethiopia’s electricity grid relies heavily on hydropower, supplying over 80% of generation but causing supply imbalances, voltage instability, and frequency deviations, especially in dry seasons. Rapid industrialization and urbanization further strain the system, making its seasonal dependence a major vulnerability. Improving voltage profiles and reducing losses are key challenges in Ethiopia’s weakly interconnected distribution networks. Unlike studies based on IEEE test feeders, this research uses real data from the Wolaita Sodo distribution system, including actual topology, load demand, conductor parameters, and operational constraints. Such a real-world context makes the findings highly relevant to Ethiopia, where utilities face significant technical and economic pressures to enhance grid performance. The application of simultaneous network reconfiguration and distributed generation (DG) placement using PSO demonstrates substantial improvements: active power loss reduction of 72.064%, voltage deviation reduction from 24.63% to 4.5%, and minimum bus voltage improvement from 0.7537 pu to 0.9550 pu. Beyond technical gains, the method delivers annual financial savings of 16.3956 million ETB with a payback period of just six years, underscoring its economic viability. By leveraging real Ethiopian grid data and integrating both technical and financial considerations, this study offers a robust and replicable decision-support framework for improving reliability, efficiency, and sustainability in emerging power systems.

## 5. Conclusion

This study evaluates a combined approach of network reconfiguration and distributed generation (DG) installation on the Wolaita Sodo feeder, modeled as an unbalanced three-phase system. Simulations using Particle Swarm Optimization (PSO) show that the integrated method outperforms individual techniques by reducing active and reactive power losses by 1175.44 kW and 59.36%, respectively, while improving the minimum voltage profile to 0.9550 pu. Economically, with a DG capacity of 4.88 MW, the strategy offers annual savings of 16.40 million birr against an investment of 22.07 million birr, ensuring a feasible six-year payback period. Overall, the findings confirm that simultaneous network reconfiguration and DG integration provide a practical and cost-effective solution for improving distribution system performance and supporting sustainable energy management. The combined reconfiguration and DG integration reduced losses by 72% and improved voltage profiles by 20%. Beyond technical gains, the practicality and scalability of the proposed method make it a suitable candidate for integration into utility operations, especially when supported by modern automation, monitoring, and protection systems. Future work will explore on dynamic reconfiguration under varying load/generation, Integration of energy storage systems, field validation in partnership with utilities, real-time implementations, and hybrid optimization frameworks for larger, more complex networks
